# Comparative efficacy and safety of imrecoxib versus celecoxib: a systematic review and meta-analysis

**DOI:** 10.3389/fphar.2025.1707079

**Published:** 2026-01-05

**Authors:** Xian Zeng, Lilin Dai, Zude Li, Xiaomin Dong, Zengzhen Liao, Liji Chen, Yuan Tan, Wei Chen

**Affiliations:** 1 Department of Pharmacy, Affiliated Hospital of Guilin Medical University, Guilin, China; 2 Faculty of Public Administration, Guilin University of Technology, Guilin, China; 3 School of Pharmacy, Guilin Medical University, Guilin, China; 4 Department of Neonatology, Affiliated Hospital of Guilin Medical University, Guilin, China

**Keywords:** imrecoxib, celecoxib, cox-2 inhibitors, meta-analysis, analgesic, safety

## Abstract

**Objective:**

This meta-analysis aimed to compare the analgesic efficacy, anti-inflammatory effects, and safety profiles of the selective cyclooxygenase-2 (COX-2) inhibitors imrecoxib (IMR) and celecoxib (CEL), providing evidence-based guidance for clinical drug selection.

**Methods:**

A systematic search was conducted of English and Chinese databases through August 2025 to identify randomized controlled trials (RCTs) comparing IMR and CEL. Methodological quality was assessed using the Cochrane Risk of Bias (ROB 2.0) tool, and quantitative analyses were performed using R software. Primary outcomes included clinical response rate, pain intensity assessed by the visual analog scale (VAS), and the overall incidence of adverse events (AEs). Secondary outcomes focused on serum inflammatory markers (CRP and ESR) and disease activity (BASDAI) in patients with axial spondyloarthritis (axSpA).

**Results:**

Pooled analyses found no significant differences between IMR and CEL in clinical response or pain reduction, indicating comparable analgesic efficacy. The overall safety profiles of the two drugs were also similar. Notably, in the osteoarthritis (OA) subgroup, IMR was associated with a significantly lower incidence of adverse events compared with CEL. An exploratory subgroup analysis in axSpA patients suggested that IMR may offer potential advantages over CEL in improving inflammatory markers and disease activity. However, this finding is based on a few small trials and should be interpreted with caution due to the low certainty of evidence.

**Conclusion:**

IMR demonstrated comparable efficacy and overall safety to CEL, supporting its role as a viable alternative selective COX-2 inhibitor in clinical practice. IMR may have potentially favorable anti-inflammatory effects in axSpA. The observed anti-inflammatory advantage of IMR in axSpA remains to be confirmed. Given the limitations of small sample sizes, short follow-up durations, and incomplete safety reporting, further large-scale, high-quality RCTs are warranted to validate these findings.

**Systematic Review Registration:**

identifier CRD420251243032.

## 1 Introduction

Osteoarthritis (OA), axial spondyloarthritis (axSpA), and other chronic inflammatory disorders are major causes of disability worldwide, substantially impairing patients’ quality of life (Abramoff and Caldera, 2020; Rasouli et al., 2022; Sieper and Poddubnyy, 2017). Non-steroidal anti-inflammatory drugs (NSAIDs) are widely recommended as first-line symptomatic treatments due to their well-established analgesic and anti-inflammatory properties (Kolasinski et al., 2019; Cañete et al., 2025; Erlenwein, 2016). By inhibiting cyclooxygenase (COX) enzymes and reducing prostaglandin synthesis, NSAIDs effectively relieve inflammation and pain.

However, long-term use of non-selective NSAIDs increases gastrointestinal risks, prompting the development of COX-2 selective inhibitors such as CEL and IMR, which provide comparable analgesia with fewer gastrointestinal adverse effects. While COX-2 inhibitors reduce gastrointestinal toxicity, concerns have emerged regarding their cardiovascular safety, as seen with the withdrawal of rofecoxib in 2004 (Ribeiro et al., 2022; Antman et al., 2007; Harirforoosh et al., 2013).

To mitigate these risks, Chinese researchers proposed a “COX-1/COX-2 balanced inhibition” approach, aiming for effective anti-inflammatory activity with improved cardiovascular and gastrointestinal safety. Imrecoxib, developed under this principle, demonstrates moderate COX-1/COX-2 selectivity (IC_50_ ratio 6.39, about 77% of celecoxib) and lacks the sulfonamide group, offering an option for patients with sulfonamide allergies (Wa et al., 2024).

Although several randomized controlled trials (RCTs) have compared IMR and CEL, most were small, single-center studies with limited methodological rigor (Zhang et al., 2023; Guo et al., 2023; Guo et al., 2022). As a result, current evidence remains inconclusive regarding their relative efficacy and safety.

Therefore, this systematic review and meta-analysis was conducted to quantitatively compare IMR and CEL in terms of analgesic efficacy, anti-inflammatory effects, and safety outcomes, providing evidence-based guidance for clinical decision-making in inflammatory disease management.

## 2 Materials and methods

### 2.1 Data sources and search strategy

Two researchers systematically searched major online databases, including: Embase (2000–2025), PubMed (1966–2025), China National Knowledge Infrastructure (CNKI, 1980–2025), Chongqing VIP Chinese Science and Technology Periodical Database (VIP, 1989–2025), Chinese Biomedical Literature Database (CBM, 1978–2025). Search terms: imrecoxib, celecoxib, pain, osteoarthritis.

### 2.2 Inclusion criteria

Only RCTs were eligible. Participants required clinically confirmed indications for IMR or CEL (e.g., osteoarthritis, traumatic injury). Studies must include an intervention group receiving IMR treatment and a control group receiving CEL treatment. Studies must explicitly describe participant selection, randomization procedures, and control methodologies. Quality assessment was conducted using the Cochrane Risk of Bias Tool. Outcome Measures: Mandatory reporting of ≥1 post-interventional outcome: pain intensity; serum inflammatory biomarker levels; clinical response rate; incidence of AEs. All included publications were restricted to English and Chinese languages.

### 2.3 Exclusion criteria

Observational studies, case reports, non-RCT experimental designs, reviews, conference abstracts, gray literature, unpublished studies and commentaries were excluded. Studies involving non-indicated conditions or animal models were excluded. Studies lacking direct IMR/CEL comparators or head-to-head comparison were excluded. Publications missing predefined outcome measures or inadequate quantitative data were excluded.

### 2.4 Study selection process

Two investigators independently performed the literature screening in two phases. Initial Screening: Titles and abstracts were evaluated against the eligibility criteria. Studies meeting inclusion/exclusion criteria were retained, while others were excluded. For duplicate publications referencing the same trial, only the dataset with the most complete outcome reporting was retained. Full-Text Screening: Potentially eligible studies underwent full-text review for final eligibility determination. Following independent screening, investigators cross-verified their selections. Discrepancies were resolved through discussion. Persistent disagreements were adjudicated by a third reviewer.

### 2.5 Data extraction

Two investigators independently extracted data using a predefined extraction template, including: Bibliographic information; Study characteristics; Intervention protocols; Outcome measures. Following independent extraction, investigators performed cross-verification. For ambiguous/inaccessible data: Attempts were made to contact corresponding authors for clarification; Studies were excluded if complete data remained unobtainable after ≥2 contact attempts.

### 2.6 Quality assessment

The Cochrane Risk of Bias Tool 2.0 (RoB 2.0) was applied to evaluate methodological quality of included studies. Two independent reviewers assessed five domains: (1) Randomization process; (2) Deviations from intended interventions; (3) Missing outcome data; (4) Measurement of the outcome; (5) Selection of the reported result. Each domain was rated as “Low risk”, “Some concern of risk”, or “High risk” of bias. Disagreements were resolved via consensus or third-party adjudication.

### 2.7 Outcome selection for quantitative synthesis

Given heterogeneity in reported outcomes across studies, primary outcome prioritization was determined by reporting frequency. Accordingly, the predefined primary outcomes for this meta-analysis are: Clinical response rate (efficacy); Pain intensity (VAS); Incidence of AEs. Secondary outcomes comprise: Post-intervention serum biomarkers: CRP; ESR; BASDAI (restricted to studies of axial spondyloarthritis).

### 2.8 Statistical analysis

All meta-analyses were conducted using R software. For continuous outcomes, treatment effects were expressed as pooled mean differences (MDs) with 95% confidence intervals (CIs). Dichotomous outcomes were analyzed using risk differences (RDs). Results were visualized via forest plots. Heterogeneity was assessed using I^2^ statistics and Cochrane’s Q-test. An I^2^ < 50% or *p* > 0.05 indicated acceptable homogeneity, warranting a fixed-effects model (Mantel-Haenszel method). Significant heterogeneity (I^2^ ≥ 50% or *p* ≤ 0.05) prompted use of a random-effects model (DerSimonian and Laird method). Sensitivity analysis was performed via leave-one-out sequential exclusion to identify influential studies. Publication bias was evaluated using Egger’s linear regression test and Begg’s rank correlation test, with results visualized in funnel plots. All effect estimates were considered statistically significant at two-sided *p* < 0.05.

## 3 Results

### 3.1 Literature search results

**FIGURE 1:**
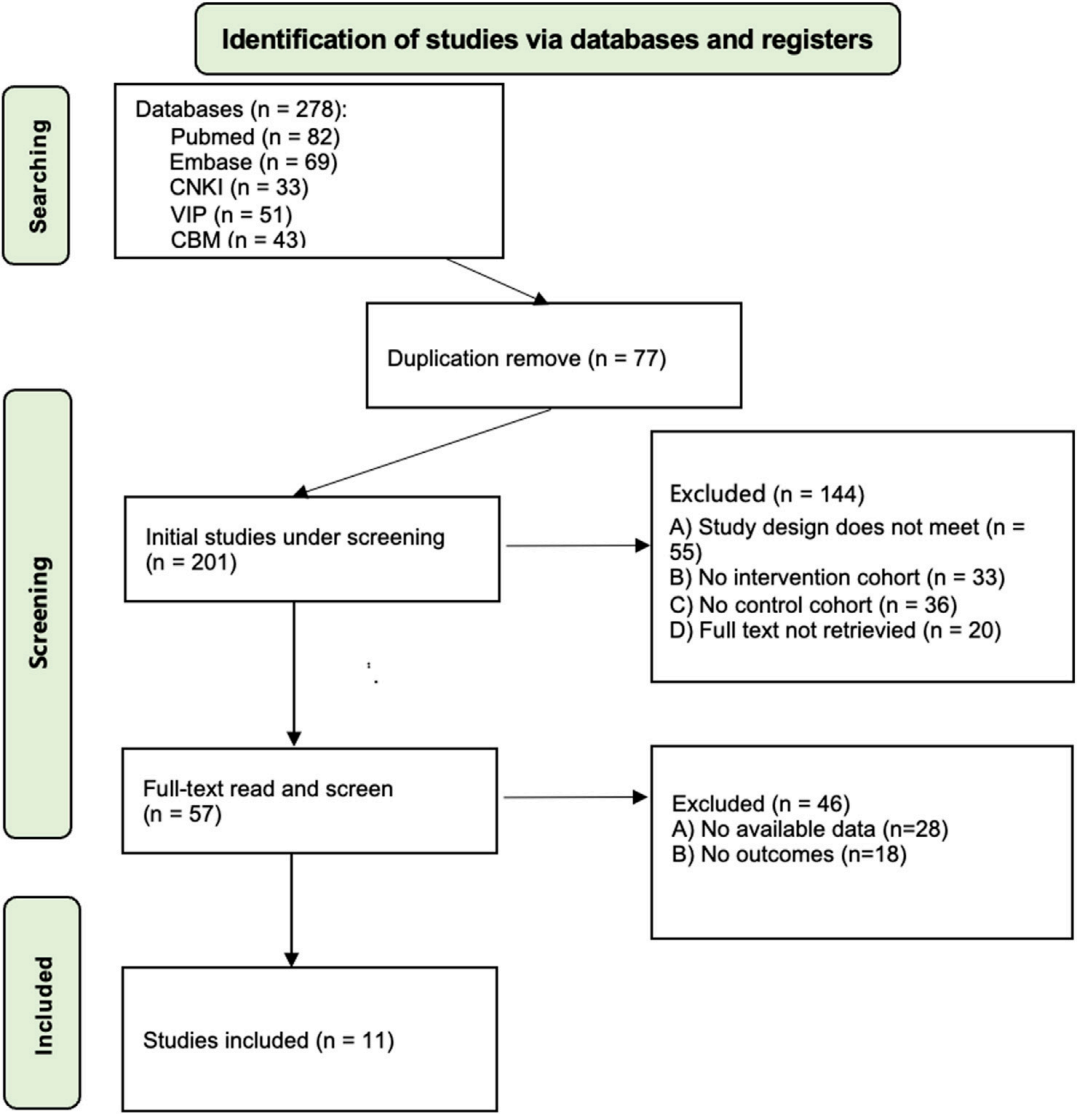
PRISMA flow diagram of study selection.

The initial search yielded 278 records. After duplicate removal and implementation of inclusion/exclusion criteria, 57 studies underwent full-text screening. Following exclusion of 46 publications owing to insufficient outcome data or incomplete metrics, 11 studies were included for quantitative synthesis. The selection process is detailed in Figure 1.

### 3.2 Characteristics of included studies

This meta-analysis incorporated 11 RCTs, collectively enrolling 1,511 participants. The intervention group comprised 873 patients administered IMR, while the control group included 638 patients receiving CEL therapy. Sample sizes across individual studies ranged from 36 to 469 participants. Publication years spanned from 2011 to 2023. The analysis encompassed trauma surgery patients in 4 trials, axSpA in 4 trials, and knee osteoarthritis in 3 trials. Detailed baseline characteristics are presented in Table 1.

**TABLE 1:** Summary of baseline characteristics, interventions, follow-up, and outcomes in included studies.

Ref.	Author	Total sample	Age	Male (n, %)	Diagnosis	IMR/CEL	IMR	CEL	Follow-up period	Primary outcomes
Zhang et al. (2023)	Zhang K et al., 2023	156	69.4 ± 5.8	53.8%	Postoperative of hip osteoarthritis	78/78	200 mg bid	200 mg bid	POD 7	Pain,AEs
Gao et al. (2017)	Gao GM et al., 2017	120	30.09 ± 8.11	75.8%	axSpA	60/60	200 mg bid	200 mg bid	12w	CRP,ESR,BASDAI,AEs
Xu (2020)	Xu YX et al., 2020	60	66.75 ± 1.78	50%	axSpA	30/30	100 mg bid	200 mg bid	12w	efficacy,AEs
Wang et al. (2020)	Wang Y et al., 2020	64	31.21 ± 6.43	66.6%	axSpA	32/32	100 mg bid	200 mg bid	12w	CRP,ESR, BASDAI,AEs
Zhang et al. (2018)	Zhang FX et al., 2018	36	60.89 ± 3.81	63.9%	axSpA	18/18	200 mg bid	400 mg bid	12w	BASDAI, pain,AEs
Xu et al. (2014)	Xu D et al., 2018	469	40–70	Not Reported	OA	351/118	100 mg bid	200 mg bid	8w	pain, efficacy,AEs
Huang et al. (2011)	Huang JL et al., 2011	127	55. 90 ± 7. 75	19.12%	OA	64/63	100 mg bid	200 mg bid	12w	efficacy,AEs
Chen (2018)	Chen LS et al., 2018	97	40. 6 ± 3. 5	54.6%	Orthopedic Trauma Surgery Patients	49/48	100 mg bid	200 mg bid	POD 5	pain,AEs
Ye (2020)	Ye SP et al., 2020	200	58.63 ± 2.15	62.5%	OA	100/100	100 mg bid	200 mg bid	8w	pain, efficacy,AEs
Guo et al. (2022)	Guo W et al., 2022	126	46.3 ± 8.2	38.9%	arthroscopic knee surgery	64/62	200 mg bid	200 mg bid	POD 3	pain,AEs
Jiang et al. (2021)	Jiang Y et al., 2021	56	30.07 ± 12.13	57.1%	oral surgery	27/29	200 mg bid	200 mg bid	POD 1	Pain

### 3.3 Quality assessment of included studies

The risk of bias for the 9 included studies was evaluated using the Cochrane ROB 2.0 tool, with results presented in Table 2. Among them, studies (Xu, 2020; Wang et al., 2020; Zhang et al., 2018; Chen, 2018; Ye, 2020) did not describe blinding and allocation concealment, indicating potential risks. The remaining studies adopted a random grouping method and provided detailed descriptions of blinding procedures. All included studies had complete data on follow-up and loss to follow-up, clearly described the outcome assessment methods, and showed no potential for selective reporting. Overall, 5 studies were assessed as having “some concerns of risk” regarding bias risk, and 6 studies were rated as “low risk”. The overall quality of the included studies was moderate.

**TABLE 2:** Risk of bias assessment based on ROB 2.0.

Ref.	Authors and publication time	Randomization process	Deviations from intended interventions	Missing data	Outcome assessment	Selective reporting	Overall bias	Weight
Zhang et al. (2023)	Zhang K et al., 2023	Low	Low	Low	Low	Low	Low	8
Gao et al. (2017)	Gao GM et al., 2017	Low	Low	Low	Low	Low	Low	8
Xu (2020)	Xu YX et al., 2020	Low	Some concerns of risk	Low	Low	Low	Some concerns of risk	8
Wang et al. (2020)	Wang Y et al., 2020	Low	Some concerns of risk	Low	Low	Low	Some concerns of risk	8
Zhang et al. (2018)	Zhang FX et al., 2018	Low	Some concerns of risk	Low	Low	Low	Some concerns of risk	8
Xu et al. (2014)	Xu D et al., 2018	Low	Low	Low	Low	Low	Low	8
Huang et al. (2011)	Huang JL et al., 2011	Low	Low	Low	Low	Low	Low	8
Chen (2018)	Chen LS et al., 2018	Low	Some concerns of risk	Low	Low	Low	Some concerns of risk	8
Ye (2020)	Ye SP et al., 2020	Low	Some concerns of risk	Low	Low	Low	Some concerns of risk	8
Guo et al. (2022)	Guo W et al., 2022	Low	Low	Low	Low	Low	Low	8
Jiang et al. (2021)	Jiang Y et al., 2021	Low	Low	Low	Low	Low	Low	8

### 3.4 Results of meta-analysis

#### 3.4.1 Post-treatment clinical response

**FIGURE 2:**
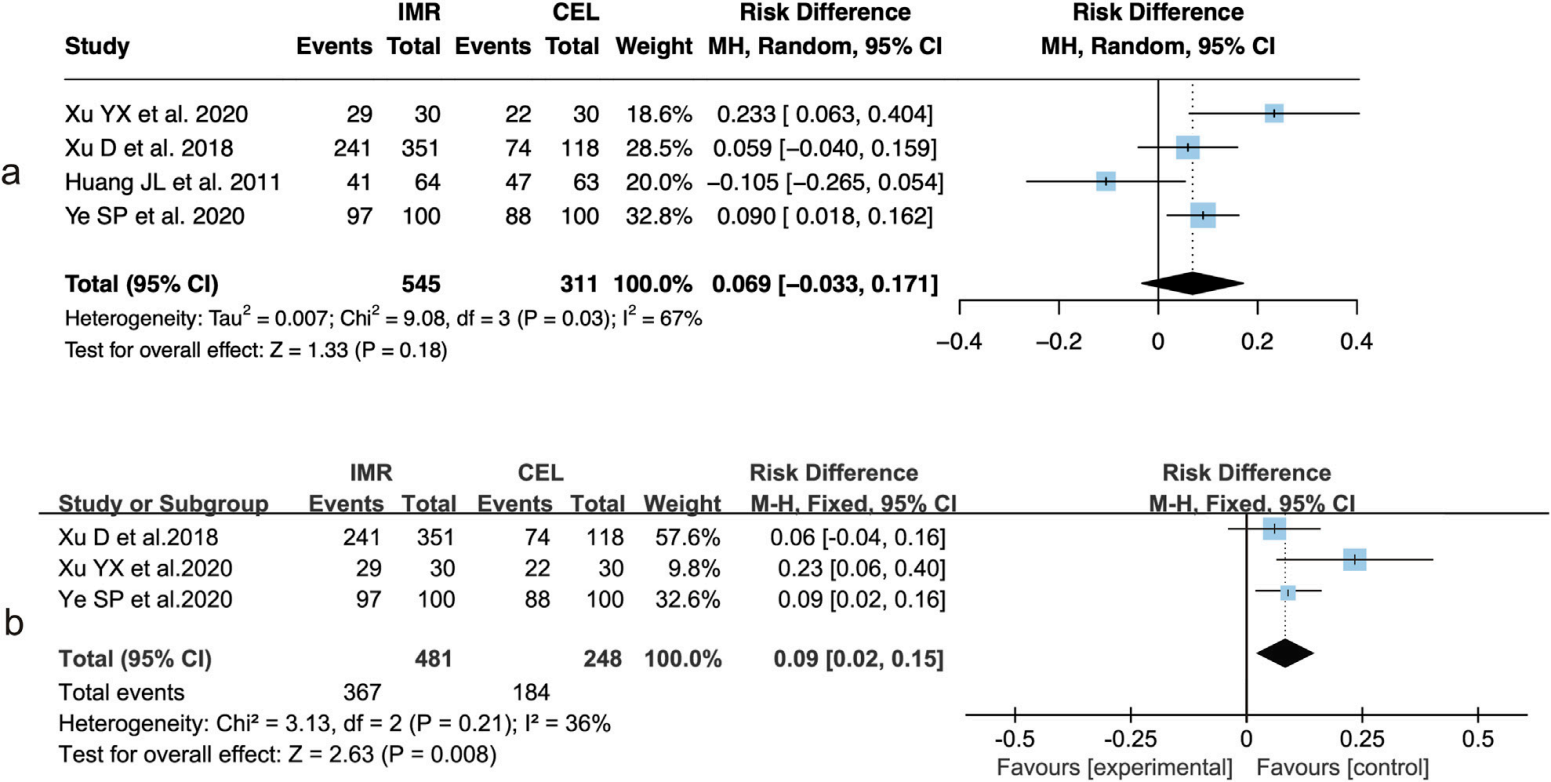
Forest plots of clinical efficacy comparisons between IMR and CEL: **(a)** Overall analysis; **(b)** Subgroup analysis in osteoarthritis (OA) patients.

In the analysis of clinical efficacy rates, four of the eleven included studies reported post-intervention outcomes (Xu, 2020; Xu et al., 2014; Huang et al., 2011; Ye, 2020). Among these, three studies (Xu, 2020; Xu et al., 2014; Ye, 2020) were conducted in patients with OA and one in patients with axSpA. The initial pooled analysis demonstrated substantial heterogeneity across studies (I^2^ = 67%, P = 0.03). Based on the random-effects model, there was no significant difference in clinical efficacy between the imrecoxib and celecoxib groups (RD = 6.9%, 95% CI: 0.033 to 0.171, P = 0.18) (Figure 2a).

A subgroup analysis limited to the three OA studies also showed considerable heterogeneity (I^2^ = 68%, P = 0.04). The pooled estimate using a random-effects model indicated that the clinical efficacy did not differ significantly between the two treatment groups (RD = 9.0%, 95% CI: 0.02 to 0.15, P = 0.008) (Figure 2b).

#### 3.4.2 VAS pain scores after treatment

**FIGURE 3:**
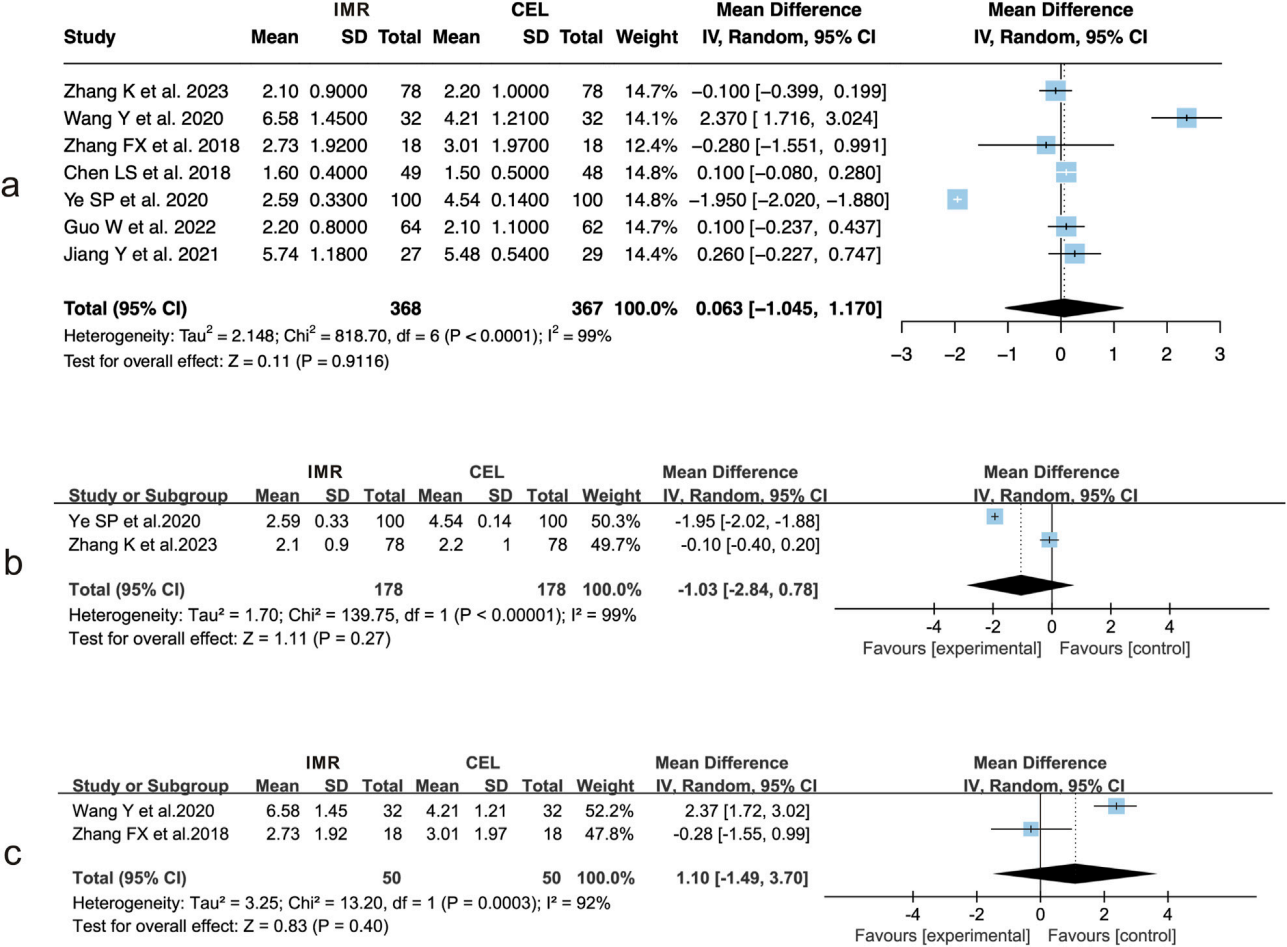
Forest plots of pain VAS score comparisons between IMR and CEL: **(a)** Overall analysis; **(b)** Subgroup analysis in OA patients; **(c)** Subgroup analysis in axSpA patients.

Among the eleven studies included, seven reporteds (Zhang et al., 2023; Guo et al., 2022; Wang et al., 2020; Zhang et al., 2018; Chen, 2018; Ye, 2020; Jiang et al., 2021) changes in pain VAS scores following intervention. These studies involved patients with OA (n = 2) (Zhang et al., 2023; Ye, 2020), axSpA (n = 2) (Wang et al., 2020; Zhang et al., 2018), as well as those undergoing orthopedic trauma surgery, knee surgery, or oral surgery (one study each). Substantial heterogeneity was observed across studies (I^2^ = 99%, P < 0.01). The pooled analysis using a random-effects model showed no significant difference in pain VAS scores between the and CEL groups (MD = 0.063, 95% CI: 1.045 to 1.170, P = 0.912) (Figure 3a).

Subgroup analyses for OA (Figure 3b) and axSpA (Figure 3c) revealed persistent heterogeneity within each subgroup. Based on the random-effects model, the differences in pain VAS scores between the two treatment groups remained statistically nonsignificant in both analyses.

#### 3.4.3 Post-treatment CRP, ESR, and BASDAI scores

Among the nine included studies, three trials (Gao et al., 2017; Wang et al., 2020; Zhang et al., 2018) specifically enrolled patients with axSpA and reported disease-specific inflammatory and activity markers, including CRP, ESR, and BASDAI. The pooled analysis showed numerically greater reductions in these markers with IMR compared to CEL (Table 3). However, given the limited number of trials (n = 3) and short treatment duration, the certainty of evidence is low. These findings suggest that IMR may have potential advantages in inflammatory control and disease activity improvement among patients with axSpA, which warrants further confirmation in larger, long-term studies.

**TABLE 3:** Effect sizes of inflammatory indicators and disease activity index indicators.

Outcomes	Reported studies	Number of reported studies	Analysis mode	I^2^ with P value	Overall combined MD	P value
CRP	Gao et al. (2017), Wang et al. (2020)	2	Random Effect Mode	0% with 0.342	2.9500 [1.9281; 3.9719]	<0.0001
ESR	Gao et al. (2017), Wang et al. (2020)	2	Random Effect Mode	0% with 0.471	3.2217 [2.2373; 4.2061]	<0.0001
BASDAI	Gao et al. (2017), Wang et al. (2020), Zhang et al. (2018)	3	Random Effect Mode	31.4% with 0.233	0.4907 [0.2125; 0.7689]	0.0005

#### 3.4.4 Comparison of post-treatment AEs

**FIGURE 4:**
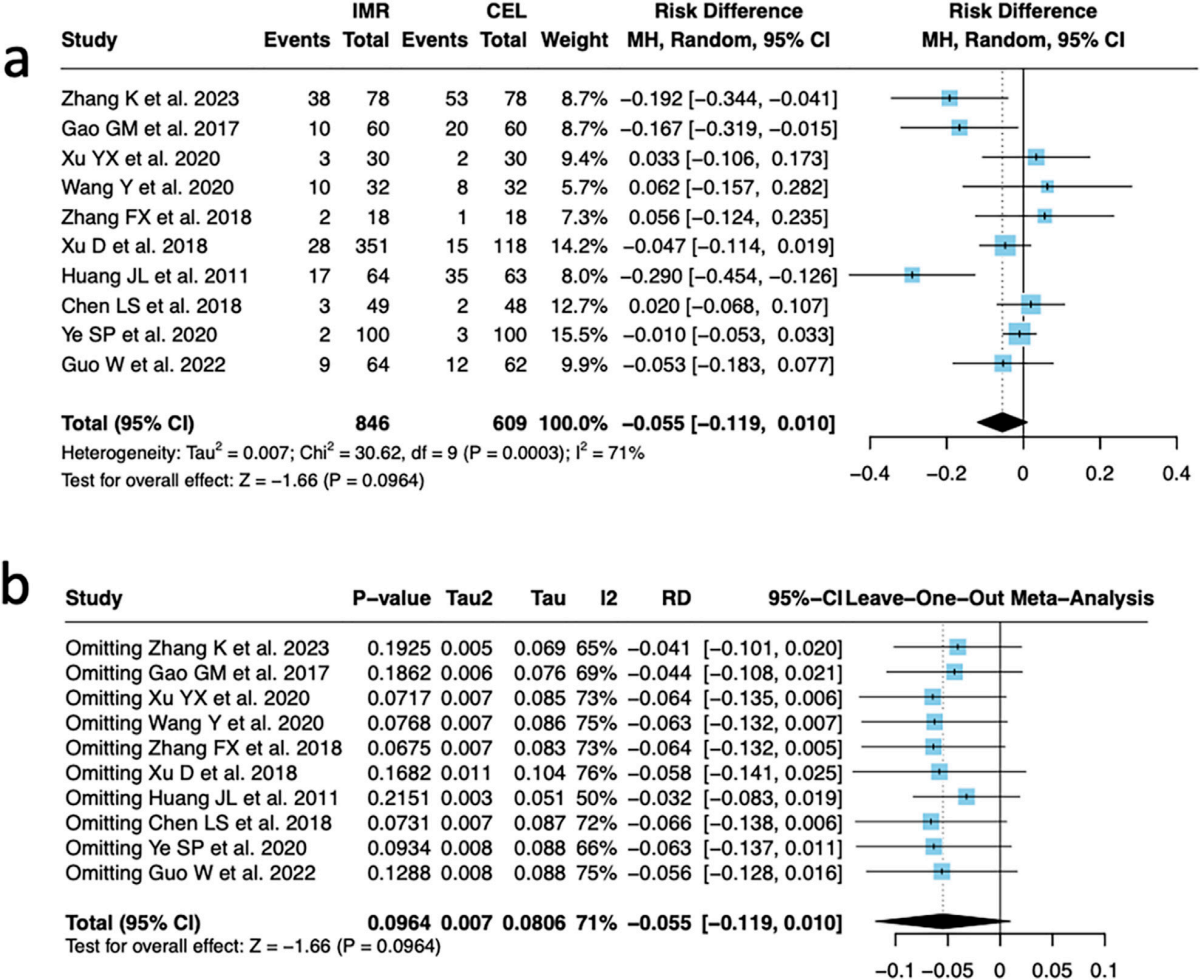
Comparison of adverse event incidence between IMR and CEL and sensitivity analysis. **(a)** Forest plot; **(b)** Sensitivity analysis.

Among the ten studies (Zhang et al., 2023; Guo et al., 2022; Gao et al., 2017; Xu, 2020; Wang et al., 2020; Zhang et al., 2018; Xu et al., 2014; Huang et al., 2011; Chen, 2018; Ye, 2020) that reported the incidence of adverse events, substantial heterogeneity was observed across studies (I^2^ = 71%, P < 0.01). Using a random-effects model, the pooled analysis showed no significant difference in the incidence of adverse events between the imrecoxib and celecoxib groups (RD = −5.5%, 95% CI: 0.119 to 0.010, P = 0.10) (Figure 4a). Sensitivity analysis was performed by sequentially excluding each study and reanalyzing the remaining nine. The results indicated that heterogeneity remained statistically significant, suggesting that all studies contributed to the overall heterogeneity, and no single study was identified as a major outlier. Moreover, exclusion of any individual study did not materially alter the direction or significance of the pooled effect, indicating the robustness and stability of the overall findings (Figure 4b).

**FIGURE 5:**
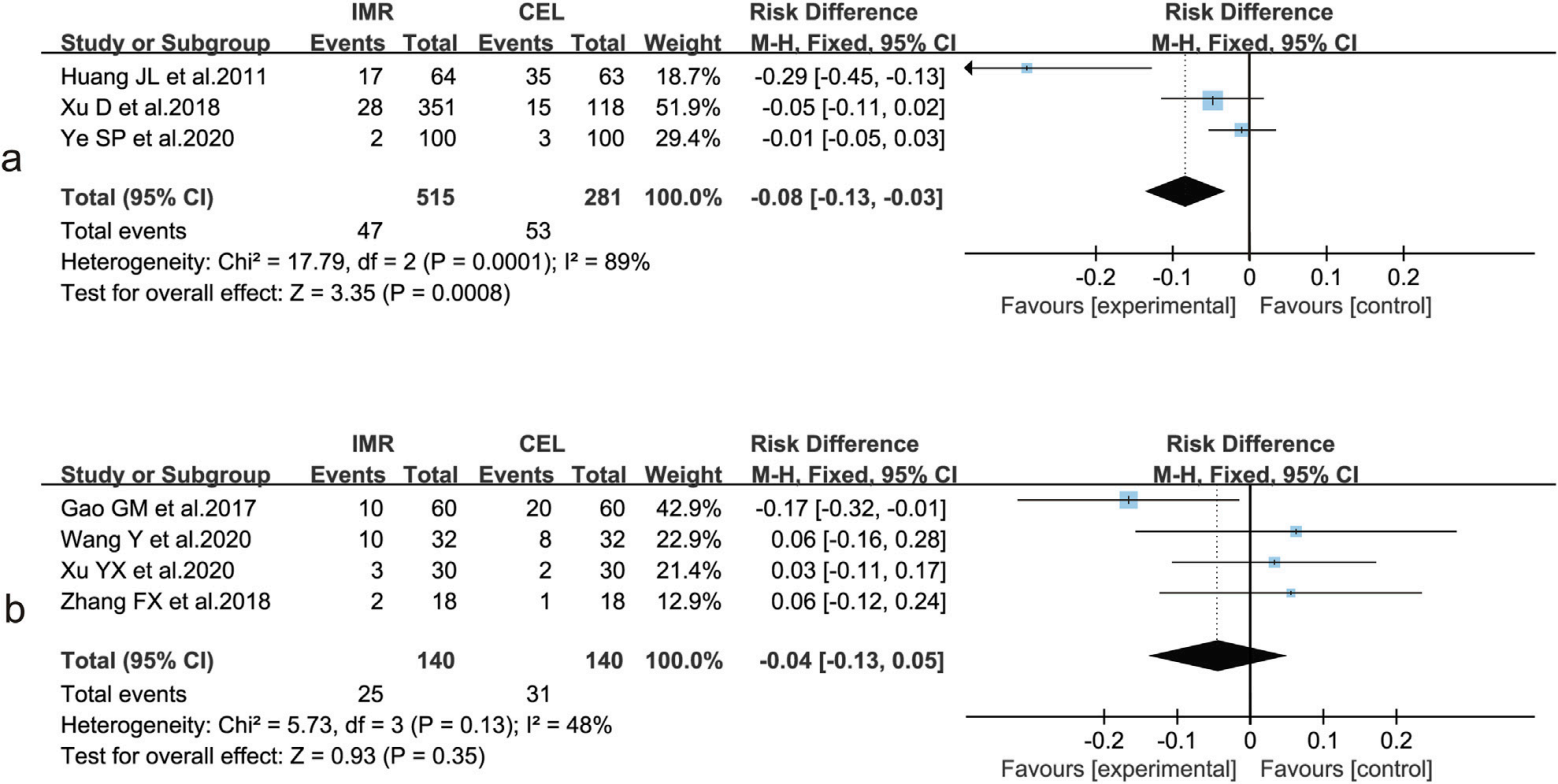
Subgroup analysis of adverse event incidence between IMR and CEL: **(a)** OA; **(b)** axSpA.

A subgroup analysis of adverse event incidence was subsequently conducted according to diagnosis, including all ten studies. These studies involved patients with OA (n = 3) (Xu et al., 2014; Huang et al., 2011; Ye, 2020), axSpA (n = 4) (Gao et al., 2017; Xu, 2020; Wang et al., 2020; Zhang et al., 2018), as well as those total hip arthroplasty, undergoing orthopedic trauma surgery, or knee surgery (one study each). Because heterogeneity within subgroups was low, a fixed-effects model was applied for both analyses. The results showed that the incidence of adverse events was significantly lower in the IMR group than in the CEL group within the OA subgroup (RD = −8%, 95% CI: 0.13 to −0.03, P = 0.0008) (Figure 5a). In contrast, the incidence of adverse events was comparable between the two treatments in the axSpA subgroup, with no statistically significant difference (RD = −4%, 95% CI: 0.13–0.05, P = 0.35) (Figure 5b).

It should be noted that, because the overall incidence of adverse events reported in most studies was relatively low and specific types of adverse events were not consistently detailed, further subgroup analyses by adverse event category could not be performed.

#### 3.4.5 Assessment of publication bias

For the outcome of adverse event incidence (number of included studies ≥10), a funnel plot was generated (Supplementary Figure S1) to evaluate potential publication bias, and Egger’s linear regression test was applied to assess funnel plot asymmetry. The results showed that the P-value of Egger’s test was 0.303 and that of Begg’s rank correlation test was 0.245, both exceeding the 0.05 significance level. These findings indicate good symmetry of the funnel plot and suggest no apparent evidence of publication bias.

## 4 Discussion

To the best of our knowledge, this study represents the first quantitative meta-analysis comparing the efficacy and safety of IMR and CEL across various pain-related and inflammatory conditions. Previous individual RCTs were limited by small sample sizes and inconsistent findings, making it difficult to draw reliable conclusions. By integrating both English and Chinese studies, this analysis provides a more comprehensive evidence base and systematically synthesizes all available data. Based on 11 randomized controlled trials involving 1,511 participants, IMR demonstrated comparable analgesic efficacy and overall safety to CEL, supporting its role as a therapeutic alternative—particularly for patients with sulfonamide hypersensitivity. Notably, exploratory subgroup analysis suggested a potential anti-inflammatory advantage of IMR in axSpA. However, this finding should be interpreted with caution and confirmed in specifically designed studies.

This study found no significant differences between IMR and CEL in terms of clinical response rate (RD = 0.069, p = 0.18) or pain improvement as measured by the visual analog scale (VAS MD = 0.063, p = 0.912). These findings are consistent with previous studies in postoperative pain and osteoarthritis populations (Zhang et al., 2023; Guo et al., 2022; Gao et al., 2017; Huang et al., 2011; Zong et al., 2022), reinforcing the non-inferiority of IMR to CEL in analgesic efficacy.

In the overall analysis, the safety profiles of IMR and CEL appeared broadly consistent, while subgroup findings in OA indicated a lower incidence of adverse events with IMR. However, given that the included RCTs involved relatively small sample sizes and did not systematically report major cardiovascular (CV) or gastrointestinal (GI) events, these results should be interpreted with caution. To provide a broader context, evidence from large real-world studies was also considered. For instance, a retrospective cohort study (Meng et al., 2021), which analyzed NSAID prescription data from 50,732 patients across three hospitals in China, suggested that IMR may be associated with a lower risk of new-onset hypertension and cardiovascular events compared with CEL in low-risk populations. Moreover, selective COX-2 inhibitors as a class were associated with a markedly reduced incidence of gastrointestinal complications compared with traditional NSAIDs. Nevertheless, observational studies are inherently subject to potential confounding factors—such as indication bias, unmeasured comorbidities, and differences in dosage or treatment duration—so these findings should be regarded as supplementary and hypothesis-generating rather than confirmatory. Overall, the safety profile of IMR warrants further validation through large-scale, prospective studies with extended follow-up.

The suggestion that IMR might possess enhanced anti-inflammatory activity in axSpA is derived from pooled results in our meta-analysis, indicating greater reductions in CRP (MD = 2.95) and ESR (MD = 3.22), alongside more substantial improvements in BASDAI scores (MD = 0.49), all with statistical significance (p < 0.01), relative to CEL. However, given the limited number of included trials (n = 3 for these outcomes: studies (Gao et al., 2017; Wang et al., 2020; Zhang et al., 2018)) and generally short treatment durations, the certainty of this evidence is low. Therefore, we have moderated our conclusion to state that “Imrecoxib may have potential advantages in inflammatory control among axSpA patients.” This finding should be considered exploratory and requires validation in larger, prospective studies with standardized inflammatory endpoints, particularly as some real-world evidence (Gao et al., 2017; Zong et al., 2022) has not observed significant differences between treatments.

This meta-analysis has several limitations. Approximately half of the included trials were rated as having “some concerns”, mainly due to unclear randomization and blinding procedures. These methodological weaknesses may have introduced performance or detection bias, thereby reducing the certainty and robustness of the pooled estimates. Although sensitivity analyses supported the stability of the findings, the influence of moderate bias cannot be completely ruled out. In addition, variations in dosing regimens (CEL: 200–400 mg/day; IMR: 100–200 mg/day) and follow-up durations (5 days–12 weeks) may have affected comparability and led to an underestimation of delayed adverse events. Incomplete outcome reporting was also noted, as only a few studies assessed inflammatory markers or major cardiovascular and gastrointestinal events, limiting the comprehensiveness of the risk–benefit evaluation. Furthermore, this analysis included only studies published in English and Chinese, which may have restricted the scope of available data and introduced potential language or publication bias. Future research should focus on large-scale, rigorously designed RCTs with standardized dosing, extended follow-up, and targeted safety evaluations in high-risk populations to strengthen the reliability of these conclusions.

## 5 Conclusion

IMR demonstrated comparable efficacy and overall safety to CEL, supporting its role as a viable alternative selective COX-2 inhibitor in clinical practice. IMR may have potentially favorable anti-inflammatory effects in axSpA. The observed anti-inflammatory advantage of IMR in axSpA remains to be confirmed. Given the limitations of small sample sizes, short follow-up durations, and incomplete safety reporting, further large-scale, high-quality RCTs are warranted to validate these findings.

## Data Availability

The original contributions presented in the study are included in the article/Supplementary Material, further inquiries can be directed to the corresponding authors.

## Supplementary material

SUPPLEMENTARY FIGURE S1 Funnel plot evaluating publication bias for adverse event incidence.
