# Supplementary figures and images for: Comparative efficacy and safety of imrecoxib versus celecoxib: a systematic review and meta-analysis
Source: Front Pharmacol. 2026 Jan 5;16:1707079. doi: 10.3389/fphar.2025.1707079 (PMC12813194; doi:10.3389/fphar.2025.1707079)

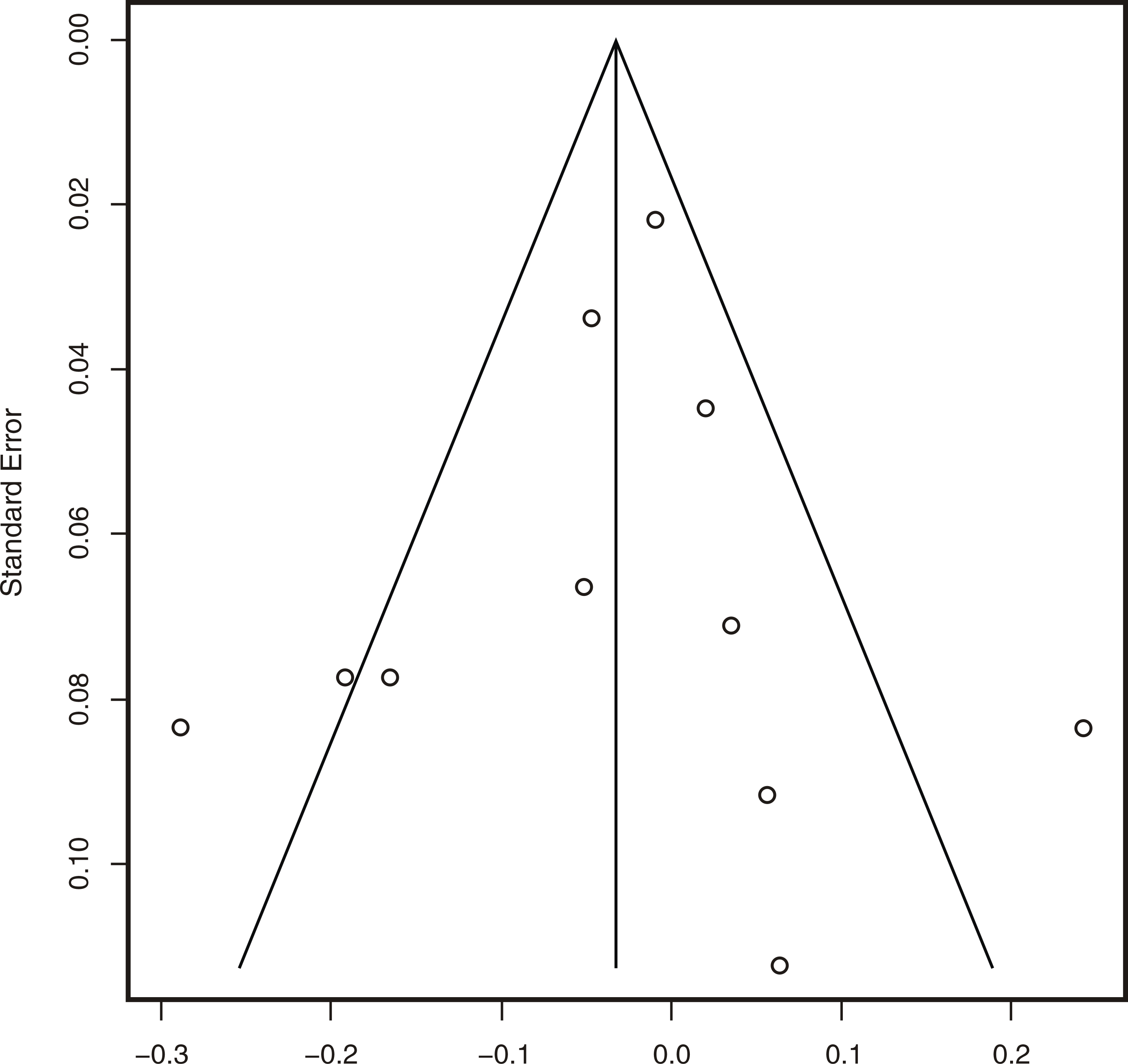

Supplement: Supplementary file 1 [file Image1.tif]
